# The Zeolitic Continuum: From Conventional Mineral Resources to Advanced Functional Materials of Bulgarian and Turkish Origin

**DOI:** 10.3390/molecules31122142

**Published:** 2026-06-18

**Authors:** Denitsa Kiradzhiyska, Kiril Gavazov, Vasil Bachvarov, Nikolina Milcheva

**Affiliations:** 1 Department of Chemical Sciences, Faculty of Pharmacy, Medical University of Plovdiv, 15A Vasil Aprilov Blvd., 4002 Plovdiv, Bulgaria; denitsa.kiradzhiyska@mu-plovdiv.bg (D.K.); kiril.gavazov@mu-plovdiv.bg (K.G.); research@s3eam.com (V.B.); 2 Research Institute, Medical University of Plovdiv, 15A Vasil Aprilov Blvd., 4002 Plovdiv, Bulgaria

**Keywords:** natural zeolites, clinoptilolite, bibliometric analysis, Bulgarian and Turkish origin

## Abstract

This review examines the scientific basis and technological development of natural zeolites, focusing particularly on deposits and research from Bulgaria and Turkey. It traces the transformation of zeolites from conventional industrial minerals into advanced, high-performance functional materials. A stepwise methodological framework was employed to conduct a bibliometric assessment of research and review articles published between 2015 and 2025, primarily focusing on contributions from researchers in Bulgaria and Turkey. In parallel, the study evaluates historical industrial data and the foundational literature. Beyond its regional focus, the bibliometric analysis reveals that Turkey ranks among the world’s leading contributors to zeolite research after Iran, China, and Indonesia, while Bulgaria maintains significant presence within the global network of active researchers in the field. The findings suggest that zeolite science in the region is expanding rapidly and dynamically, with current research increasingly focused on modifying, functionalizing, and diversifying zeolitic materials for a wide range of scientific and technological applications, including wastewater treatment, environmental remediation, construction materials production, agriculture, animal husbandry, and healthcare enhancement. The growing demand for effective adsorbents, therapeutic agents, and nutritional supplements, coupled with the critical need to reduce manufacturing costs, serves as a primary driver for accelerated zeolite research.

## 1. Introduction

Zeolites were first introduced in the 18th century by Axel Fredrik Cronstedt. Today these hydrated aluminosilicate minerals have a wide range of diverse applications, including industrial, environmental, and biomedical [1]. Their structural matrix consists of a microporous, three-dimensional framework of silicon and aluminum, stabilized by exchangeable cations (e.g., sodium, potassium, calcium, magnesium, barium or strontium). This configuration forms an interconnected system of cavities and channels which allow the minerals to trap or exchange ions and small molecules in an aqueous environment [2,3]. A large array of zeolites, encompassing over 40 natural and 230 synthetic varieties, have been already identified [1,4]. In terms of classification, this significant group of aluminosilicates is divided based on their nature, structure, pore size, Si/Al ratio, and thermal stability [5,6]. Among the zeolites found in nature are clinoptilolite, chabazite, analcime, ferrierite, laumontite, scolecite, heulandite, phillipsite, mordenite, and stilbite [5]. Variations in the mineralogical and chemical profiles of natural zeolites are linked to their specific geological environment [7]. Their porous structure, ion-exchange capability, substantial adsorption capacity, and greater chemical selectivity, compared to silica and activated carbon [8], are among the most frequently cited factors contributing to their utilization across a broad spectrum of industrial sectors [9]. Their distinctive three-dimensional network skeleton allows natural zeolites (NZ) to function as molecular sieves, thereby offering a valuable tool for separation, purification, and catalysis. To broaden their range of applications and improve their physicochemical, morphological, and structural features, various pretreatment methods have been proposed. Chemical, physical, thermal, and combined pretreatment approaches have been shown to enhance microporosity, ion-exchange capacity, removal efficiency, catalytic activity, and other functional properties, thereby optimizing their performance in specific utilizations [10].

**Figure 1:**
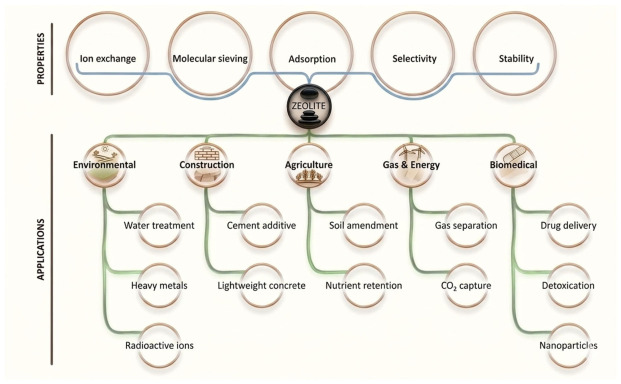
Natural zeolite feature–function relationships.

These minerals are considered highly effective in the removal of radioactive and toxic substances, and clinoptilolite remains the primary choice for various biomedical applications [11]. Some of the most valuable feature–function relationships are represented in Figure 1.

The commercial dominance of zeolites among porous frameworks and their commercialization through the years is attributed to their exceptional stability and cost-efficiency. Backed by decades of proven performance in different sectors, they continue to be a cornerstone of industrial chemistry [12]. Clinoptilolite, chabazite, and mordenite are widely implemented in wastewater treatment and environmental remediation because of their effectiveness in removing radionuclides, heavy metals, ammonium ions dyes, and surfactants [13,14]. In view of their favorable effect on soil characteristics, NZ are extensively deployed as fertilizers and amendments [15]. Additionally, in animal farming, zeolites are valued for boosting nutrient uptake and protecting livestock from harmful toxins in their diet. Along with this, as a premier solution for sustainable aquaculture, NZ provide critical benefits by improving fish nutrition, enhancing oxygenation systems, and detoxifying water of ammonium cations [9]. In terms of gas purification processes, pressure-swing adsorption is used to purify methane from landfills and remove acidic contaminants from industrial gas streams, with chabazite and mordenite being employed. Their distinctive crystalline structures act as size-selective molecular sieves, leveraging distinct diameters to facilitate the selective trapping of nitrogen, thereby enabling the production of enriched oxygen for use in hospitals, fish farming, and wastewater treatment [16]. The exceptional thermal stability and acid resistance of clinoptilolite, coupled with its high CO_2_ selectivity, make it an ideal material for carbon capture. The mineral plays a vital role in mitigating atmospheric emissions by effectively isolating greenhouse gases under harsh industrial conditions [6]. The historical and widespread implementation of NZ as pozzolanic additives within the global construction industry is well documented. Due to their reactive properties, these minerals are significant supplementary cementitious materials in many regions. In China specifically, the extent of this integration is substantial, with an annual consumption of around 30 million tons [17]. In the biomedical field, NZ are often referred to as antitoxins. By selectively adsorbing toxic cations and biomolecules, these minerals provide a sustainable solution for purifying water [8] and detoxing animal and human organisms [18]. Clinoptilolite has been licensed as an antidiarrheal medication under the name Enterex^®^ since 1995 [19]. Furthermore, an antacid medication called Neutracid^®^ primarily comprises clinoptilolite derived from the Tasajera deposit [20]. Evidence supports the role of natural zeolites as hemostatic agents. Specifically, their high porosity allows them to facilitate rapid dehydration of the wound site by absorbing water molecules to concentrate clotting factors, while simultaneously serving as oxygen carriers and biological triggers for platelets [21]. Field observations further support these healing properties; workers at several American zeolite mines have reported that incidental dust exposure speeds up the recovery of cuts and scrapes. This aligns with veterinary practices, where clinoptilolite powder is topically applied to horses and cattle to enhance wound healing [22]. Through modulating sebum production, modified clinoptilolite complexes have been reported as an effective therapeutic agent for managing oily and acne-prone skin types [23]. Additionally, clinoptilolite provides the essential calcium and silica needed for new bone growth, promoting healing after tooth extraction. Its ion-exchange capacity and absorption features detoxify the socket by trapping bacteria, histamine, and inflammatory proteins [24].

Other reported pharmaceutical applications of unmodified NZ, particularly clinoptilolite, mordenite, and faujasite, include their use as active components in formulations for gastroesophageal reflux disease, osteoporosis, cancer therapy, and antiviral treatments, as well as drug-delivery carriers for acne treatment, endometriosis, fibrocystic breast disease, and related conditions. Unmodified faujasite has emerged as an optimal framework for regulated drug release applications [11]. However, safety profiles vary across zeolite species; specifically, erionite has been definitively linked to the development of lung cancer and malignant mesothelioma [25,26]. Studies have linked chronic erionite dust exposure to high mortality rates in several Turkish villages [27].

Additionally, zeolites provide a stable, porous scaffold for nanoparticle-based drug delivery due to their unique crystalline architecture [28]. Zeolite nanoparticles emerge as powerful tools for therapeutic delivery, frequently compared to mesoporous silica nanoparticles, which are a common focus of drug release studies. The rising importance of NZ in targeted therapy has led to studies into their possible use in improving cancer treatments. Merging active drug compounds with clinoptilolite and synthetic zeolites has led to a major breakthrough in the development of human therapeutics [1,29]. Moreover, tailored zeolitic frameworks outperform natural ones by enabling advanced drug release strategies, including pH-sensitive and electromagnetic-field-driven delivery systems. Although zeolite-based nanoparticles have many advantages, significant challenges in terms of synthesis control and scalability must be overcome before they can be successfully commercialized [5].

While clinoptilolite shows significant promise in pharmacotherapy due to its proven antioxidant, anti-inflammatory, and detoxifying benefits, it is not approved for use as a food supplement in the EU. According to the Novel Foods Catalogue, the European Food Safety Authority (EFSA) has yet to officially confirm its safety for human consumption [30]. However, zeolite-based supplements are often marketed before their scientific evidence is validated [31], with drugstores promoting their “super material” status and making claims without stringent verification. While zeolites offer significant therapeutic potential, their current status in the nutritional market is sometimes subject to unsubstantiated or exaggerated health claims [18]. Such practices are particularly evident in Turkey, a non-EU country, where promotional materials frequently emphasize detoxification and broad health benefits. Similar marketing trends are also observed in Bulgaria, where websites and organic stores sell dietary supplements containing Rhodope zeolite (clinoptilolite). In response, the Bulgarian Food Safety Agency (BFSA) periodically conducts inspections, removes non-compliant products from the market, and warns consumers about “misleading labeling” [32].

Without a doubt, zeolite is positioned as one of the most commercially viable porous materials, supported by extensive academic research published over the decades [33]. While existing scientific studies often evaluate local NZ deposits, this review adopts a regional perspective by focusing on contiguous Balkan–Anatolian geological resources. Bulgaria and Turkey were selected as the primary focus due to their substantial zeolite reserves and their contribution to the development and utilization of these minerals. This study provides a comprehensive overview of how similarities in their mineralogical profiles [2], particularly the prevalence of high-purity clinoptilolite-rich deposits [34,35], support diverse technological applications and materials development pathways. Bulgaria has played a prominent role in the early commercialization of natural zeolites, whereas Turkey is characterized by extensive reserves and a broad spectrum of contemporary applications. Together, they form a distinctive geographical and functional landscape, enabling a broad examination of how regional mineral resources can evolve into the next generation of advanced functional materials.

## 2. Bibliometric Research and Analysis

For the purposes of this study, bibliometric research was conducted to provide a quantitative overview of recent scientific trends in the Scopus database from 2015 to 2025. However, this approach does not fully capture the region’s historical and material-specific context. Many international collaborations involving Bulgarian and Turkish authors utilize foreign zeolitic samples, while significant foundational data on local deposits remains contained within local or non-indexed reports. Therefore, this review adopts a broader approach than a strictly Scopus-based methodology, incorporating selected relevant works regardless of publication date or database coverage, where the investigated aluminosilicate originates from Bulgaria or Turkey. This approach ensures a coherent continuum linking the region’s natural mineral heritage with its contemporary experimental research frontiers.

### 2.1. Definition of Search Criteria and Data Collection

In order to establish the research objectives, the keywords “natural zeolite” and “clinoptilolite” were identified as primary inclusion criteria for the Scopus search. Owing to its sustainable nature, biocompatibility, widespread availability, economic viability, and the substantial body of research dedicated to its properties and applications, clinoptilolite was adopted as a primary keyword for the bibliometric search [6]. Conversely, the terms “clay” and “synthetic zeolite” were explicitly excluded to ensure specificity. This filtering process was validated by the authors’ research expertise and preliminary bibliographic reviews, which revealed the need to distinguish NZ from similar materials. To ensure data consistency and quality, this study utilized Scopus, a multidisciplinary database favored for its comprehensive collection of documents across diverse academic disciplines.

The study analyzed Scopus-indexed literature spanning from 2015 to 2025, encompassing only review and research articles published in English. Consistent with the defined criteria, the following search query was used: TITLE-ABS-KEY (“natural zeolit*”) OR TITLE-ABS-KEY (“clinoptilolit*”), AND NOT TITLE-ABS-KEY (“synthetic zeolite*”), AND NOT TITLE-ABS-KEY (“clay”). Wildcards (asterisks) were employed to capture all morphological variants of the keywords.

### 2.2. Documents and Citations

**Figure 2:**
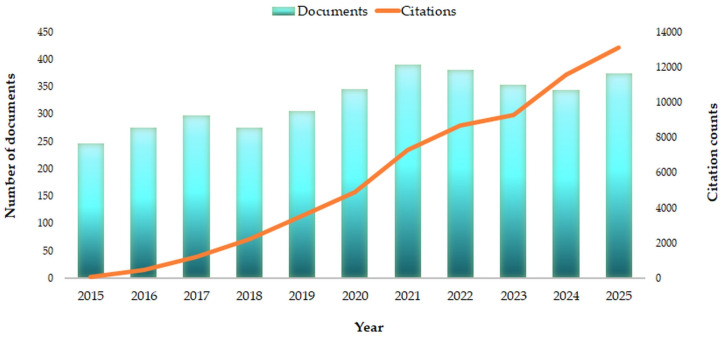
Scientific research output in Scopus database on NZ (2015–2025); number of documents and citations per year. The line indicates the citation counts.

This search strategy resulted in 3588 documents for the defined period. As illustrated in Figure 2, the annual scientific research output on natural zeolites has shown a sustained growth trend, peaking in 2021, while the cumulative citation count has experienced steady growth over the same period.

### 2.3. Geographic Distribution

**Figure 3:**
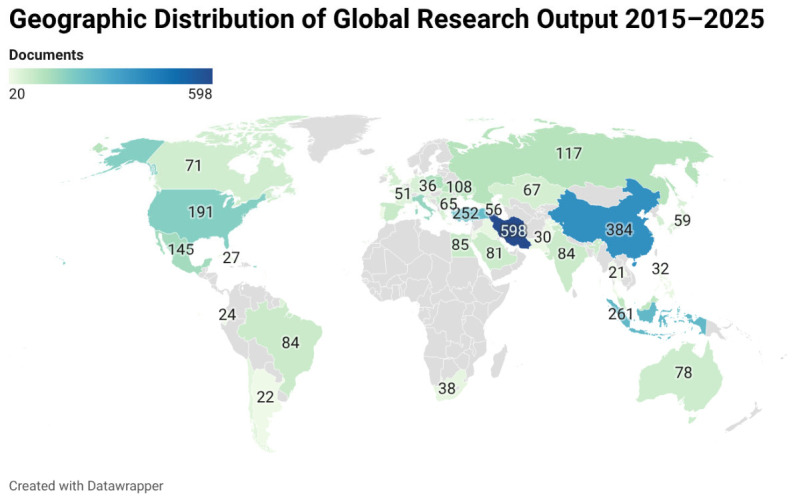
Geographic distribution of documents published in Scopus from 2015 to 2025, generated using Datawrapper GmbH (Berlin, Germany, Available online: https://www.datawrapper.de, accessed on 9 June 2026).

The geographic distribution of published documents generated using Datawrapper GmbH (Berlin, Germany) is represented in Figure 3. Following Iran, China, and Indonesia, Turkey is a leading global producer of zeolite research (with 252 publications), while Bulgaria also holds a substantial presence, ranking 25th (with 65 publications) among the contributing countries listed in the Scopus database. To be included in the global map, a minimum threshold of 20 published documents throughout the research period was required.

### 2.4. Country Co-Authorship Network

**Figure 4:**
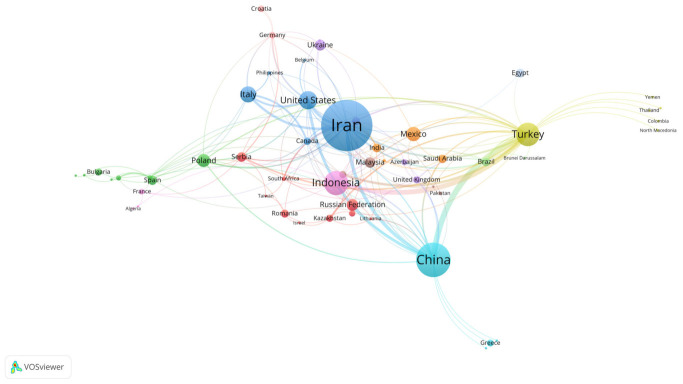
Country co-authorship map during the period 2015–2025 (generated using VOSviewer, v. 1.6.20).

Figure 4 illustrates the country co-authorship network, generated using VOSviewer (version 1.6.20). A minimum threshold of five co-occurrences was required for inclusion in the network map. The size of each connected point represents a country’s publication productivity, while the thickness of the lines reflects the strength of its international co-authorship collaborations. The map shows a highly interconnected global landscape dominated by Iran, China, Turkey, and the United States. Iran occupies a central position, acting as a collaborative connector between major Asian research contributors, such as China and Indonesia, and Western nations, such as the United States and Italy. A prominent European cluster (shown in green) also indicates a strong regional co-authorship network. Overall, these findings demonstrate that these leading countries form the primary foundation of this research field.

### 2.5. Turkey and Bulgaria Performance Analysis

**Figure 5:**
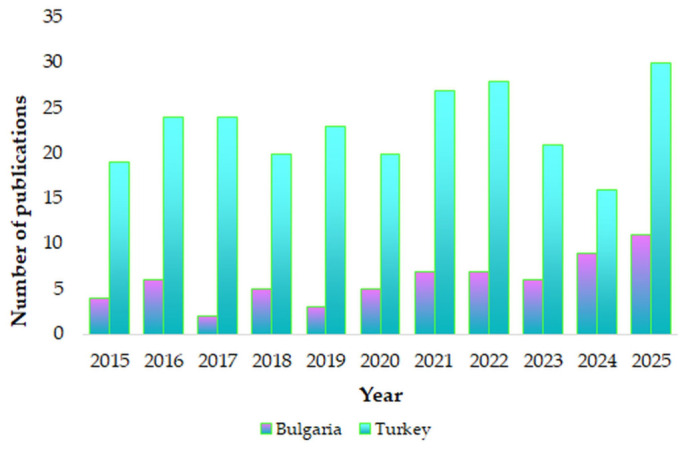
Annual scientific performance of Bulgaria and Turkey from 2015 to 2025.

The Scopus data analysis of publication performance for Bulgaria and Turkey indicates that Turkey remains the dominant contributor of zeolite assessment in the region. According to Figure 5, Turkey exhibits a more established, high-volume research profile, whereas Bulgaria has demonstrated a notable acceleration in its publication rate since 2023. The results suggest an intensifying regional focus on zeolite science during the observed period, with a predominance of contributions from Turkey owing to its larger population (13 times larger), geographic size, and substantial mineral reserves. It is noteworthy that original research papers comprise 97.2% of the combined scientific output for both countries, indicating a substantial prevalence of experimental studies over reviews.

Furthermore, the scientific impact of both countries was assessed based on the citation frequency of their most prominent authors. The most highly cited publications from the selected period are detailed in Table 1, only one of which is a review article. In the table are included the number of citations of each document in the research period (2015–2025)* and the total number of citations (up to May 2026). The data reflects a dynamic shift in regional zeolite science, as investigators focus on structural modifications and the expansion of these minerals’ utility across diverse scientific fields.

## 3. The Influence of Geological Origin on the Properties and Applications of Zeolites

The industrial and environmental performance of natural zeolites is fundamentally governed by their geological sourcing and the specific exchangeable cations that are inherently present within their structural composition. The physical–chemical properties of NZ are inextricably linked to their geological deposits [45]. Geographically, these deposits are typically grouped into European, Asian, African, and North American classifications. This relationship is clearly demonstrated in comparative studies of various regional deposits. For instance, a comparative analysis of European clinoptilolites indicates that Greek and Slovakian zeolites generally outperform Bulgarian varieties in ammonium and orthophosphate removal. While Greek samples exhibit high ammonium uptake, specific Slovakian variants demonstrate a uniquely balanced dual functionality for both ions. In comparison, Romanian clinoptilolite shows moderate ammonium adsorption. Asian zeolites, primarily represented by Chinese and Turkish deposits, also show significant efficacy. Heulandite-derived minerals and those sourced from Chinese lake sediments display high adsorption capacities, which can be further enhanced through chemical modifications like sodium nitrate calcination. Ultimately, these performance disparities are driven by the Si/Al ratio. This ratio, determined by the zeolite’s specific geographic source and geological formation, dictates the framework’s negative charge and subsequent ion-exchange capacity [46]. The specific type and quantity of ions already present in the structure have been demonstrated to be linked to the aluminosilicates’ ability to uptake new contaminants. For example, the presence of specific cations determines the theoretical cation-exchange capacity and the order of selectivity for heavy metals [47]. Pre-existing ions can also facilitate or hinder specific modifications. Research into silver nanoparticle formation showed that Na-modified NZ outperformed H-modified form in antibacterial activity assessments [48]. These observations demonstrate how the geographical origin of minerals and raw material composition modulates their success in subsequent applications.

**Figure 6:**
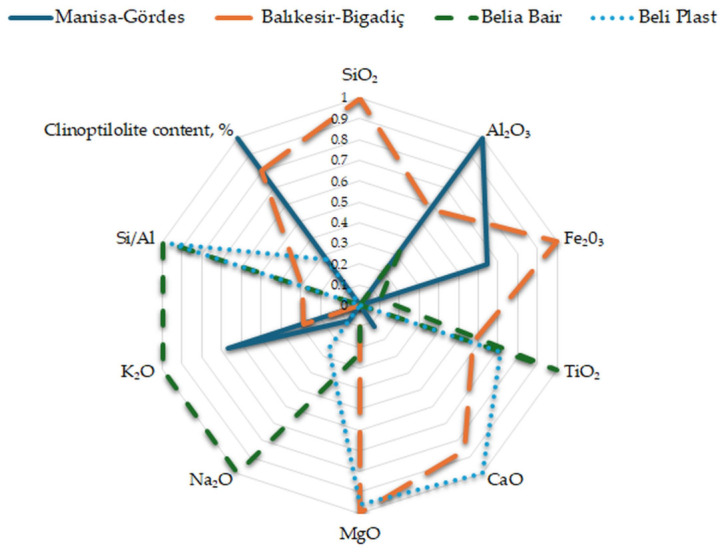
Comparative analysis of normalized NZ compositions from different regions of Bulgaria and Turkey.

The inherent chemical compositions and mineralogical purity of these zeolites vary significantly across different geographical regions. Figure 6 illustrates the comparative compositional data for deposits in Bulgaria and Turkey, as reported by [49] and [35], respectively.

The divergence in material properties based on origin is clearly visualized in the comparative geochemical profile presented. The Turkish zeolites, particularly from the Manisa-Gördes region, exhibit the highest clinoptilolite purity and alumina content. Conversely, the Bulgarian samples from Beliya Bair and Beli Plast are characterized by higher Si/Al ratios and a distinct enrichment in alkaline earth metal oxides like CaO and MgO. These disparities suggest that the Turkish samples may potentially be more suitable for applications requiring high ion-exchange capacity, while Bulgarian samples offer a distinct structural stability that is modulated by their specific geological origin.

The chemical profiles provide the quantitative context for the subsequent analysis of the Turkish and Bulgarian zeolite continuum, as documented through various regional studies and characterization efforts that establish the industrial potential of the local deposits.

### 3.1. Zeolitic Resources of Turkey

The geographical distribution of zeolite mineral formations across Turkey is diverse, spanning from Edirne to Sivas. Turkey’s zeolite resources consist predominantly of clinoptilolite, with total deposits estimated at roughly 50 billion tons. The primary, high-grade, and industrially significant clinoptilolite-rich deposits are in Gördes (Manisa Province) and Bigadiç (Balıkesir Province) [50]. Some additional regions which are zeolite-bearing, but do not contain high-grade clinoptilolite, are the Kütahya region, Çankırı–Çorum Basin, and Demirci (Manisa) [51]. Other specialized minerals, including analcime, mordenite, and chabazite, are also present, often occurring in combination within specific locales like Ankara and Nevşehir [50,52].

Although zeolites were commercialized globally in the 1960s, they were subsequently identified in Turkey in 1971 [53,54]. During this period and into the early 1980s, extensive studies on Turkey’s natural zeolite deposits were carried out, focusing on their identification, quantification, and industrial application [55].

Given that a major global application of natural zeolites is the removal of radioactive isotopes, Turkish minerals—specifically zeolites and clays—have been extensively researched for their radionuclide adsorption capabilities. Additional investigations have examined diatomites, volcanic tuffs, and perlites for the removal of elements such as strontium, cesium, and uranium. These studies utilize both inactive isotopes and radioactive tracers. Examining the influence of parameters such as pH and temperature on adsorption isotherms, kinetics, and thermodynamics establishes the sequestration potential of these local minerals [50].

The availability of approximately 4.5 million tons of high-quality clinoptilolite in Turkey has provided a significant incentive for its large-scale adoption in industrial wastewater treatment processes [56]. Though numerous removal experimental studies have investigated the utilization of NZ, every specific zeolite material requires individual research to determine its unique adsorption characteristics and performance [57]. In Table 2 are listed selected research studies involving samples of Turkish origin.

Despite their reputation for trapping toxins and radioactive waste, zeolites can act as a radiation source due to their volcanic origins. Studies have determined the activity concentrations of ^226^Ra, ^232^Th, and ^40^K in building materials and evaluated the radiological risks of their use [73,74]. Furthermore, a comparison of the radiological impact of NZ from Greece, Serbia, and Turkey as soil conditioners, dietary supplements, and construction materials was also investigated by evaluating external radiation doses, intake, and indoor exposure. Potential physicochemical mechanisms were explored through correlation analyses of ^238^U, ^226^Ra, ^210^Pb, ^228^Ra, ^228^Th, and ^40^K. The study defined environmental application limits by testing various zeolite proportions in bricks and cement. Radiological assessments indicate negligible risk from using zeolites as soil amendments and dietary supplements. In contrast, applying these minerals in construction materials results in a more significant radiological footprint [75]. Concurrently, another evaluation was conducted on the safety of Western Anatolian zeolites intended for utilization in the construction, agricultural, and chemical industries. The presence of measurable quantities of ^226^Ra, ^232^Th, and ^40^K in these samples was reported to remain within permissible limits [76].

In accordance with global sustainability goals, enhancing the nitrogen loading capacity of natural zeolites offers an efficient alternative to conventional fertilizers. These enriched minerals improve soil health by modulating nutrient delivery to meet specific plant demands. Consequently, this results in a reduction in the total chemical footprint within agricultural ecosystems. Experimental study into sustainable agriculture has highlighted the efficacy of zeolite-based fertilizers, specifically those synthesized through the adsorption of nutrients into porous mineral matrices. Utilizing clinoptilolite from the Gördes region of Turkey, nitrogen-enriched fertilizer was developed by combining it with NH_4_NO_3_ [77].

Tracing back the broader scientific interest in local minerals, during the 1980s and 1990s, Turkish researchers conducted experiments to ascertain the capacity of their domestic NZ to reproduce the promising outcomes of animal dietary zeolites documented in international studies. Numerous experimental investigations considered the improvement of growth performance, impact on electrolyte balance, antidiarrheal infection prevention, beneficial effect on bone mineralization and calcium and phosphorus utilization, and metabolism in living organisms. The discrepancies in the results reported are attributed to various factors, including the type of mineral, its purity, physicochemical properties, and the level of supplementation [78]. Beyond these physiological benefits, clinoptilolite has been proposed as a protective agent against mycotoxin-induced toxicity in animal models [79]. In Sprague-Dawley rats, dietary supplementation with Manisa clinoptilolite improved the digestibility of most nutrients without affecting relative organ weights, although fiber and crude ash digestibility were reduced. Collectively, these findings support the use of Manisa zeolite as an effective natural toxin binder in animal feed when proper storage conditions are maintained [80]. Beyond its role in systemic nutrition, the therapeutic application of this mineral extends to specialized veterinary care. For instance, the combination of clinoptilolite with amoxicillin-clavulanic acid offered a promising tool for veterinarians managing canine periodontitis. The study indicated that this modified material improved clinical signs while simultaneously reducing tissue inflammation and bone loss. These findings support the use of this combination as an effective protocol for treating dogs with advanced periodontal inflammation [81].

Alongside these experimental frameworks, commercial zeolite additives today (Nat Min 9000^®^, Gördes Zeolit Madencilik San. ve Tic. A.Ş. (Öztürk Group), İzmir, Manisa, Turkey) originate from the Gördes region of Manisa, Turkey, which holds one of the world’s largest and purest clinoptilolite deposits. Extensive research has been conducted on the efficacy of this material as a feed additive and filter medium for freshwater aquariums [82], on heavy metal levels in milk [83], in the diets of fattening pigs [84], in horse feed [85], on weight gain in Holstein calves [86], etc. Additionally, Gördes zeolite is currently a commercial material employed in soil conditioning, animal bedding, water filtration, and the manufacturing of cat litter. Moreover, several Turkish manufacturers provide standardized zeolite products that are frequently implemented in the agricultural and veterinary sectors.

Recently, Turkey developed a sophisticated industrial infrastructure to transform its high-purity deposits into specialized agricultural solutions. This industrial capacity supports the production of advanced toxin binders such as Vitatox^®^, Nutri Toxin Binder^®^, and Retox Power^®^, which are specifically engineered to mitigate mycotoxin-related risks in livestock. These products prevent the absorption of toxins into the bloodstream, thereby protecting animal performance and health. The safety and efficacy of these clinoptilolite-based additives are underscored by rigorous regulatory standards, ensuring they meet the technical specifications required for animal nutrition and feed safety [87].

### 3.2. Zeolitic Resources of Bulgaria

The formation of zeolitic rocks in Bulgaria is associated with sedimentation in intramountain grabens resulting from collisional volcanism. The rocks are classified into three genetic types: geoautoclave type, hydrothermal type, and redeposited clinoptilolitic rocks. Clinoptilolite is identified as being of primary origin, meaning it was already formed within the volcanic fragments before they were eroded and redeposited into new sedimentary layers. Its content in NZ rocks varies between 10% and 80 wt. % but is usually about 50% [88]. The largest zeolite deposits explored in the country are Beli Plast, Gorna Krepost, Most, Golobradvo, and Beliya Bair for clinoptilolite and Lyaskovets for mordenite [89].

Between 1976 and 1986, the focus of Bulgarian researchers was on comprehensive study in the field of sourcing and evaluating natural raw materials. Zeolitized tuff from Zhelezni Vrata, known as trass, has served as a standard component in the cement industry for over 75 years. The addition of NZ in cement pastes significantly depletes portlandite content through pozzolanic activity, reaching total exhaustion in specific additive-heavy blends [90,91].

Bulgaria occupies a notable place in the history of extraterrestrial agriculture, having become the third country in the world, after the USA and Russia, to develop a functional space greenhouse. At the “Zeolite Meeting ’95” in Sofia, researchers presented a landmark report called “Zeolite Gardens in Space”, which was built on research by the US Air Force and Soviet scientists from 1950 to 1980 [92]. The foundation of this achievement is the renowned zeolite-derived substrate known as Balkanin. Developed and patented in 1979, it was engineered as a high-performance substitute for conventional soil. Using thermally and chemically activated clinoptilolite, this “ion-exchange soil” provides an ideal feeding environment for plants in a variety of settings, ranging from terrestrial greenhouses and open fields to environments in low Earth orbit [93]. In 1984, a joint Bulgarian–Russian team created the SVET space greenhouse, which utilized the advanced substrate. The system’s full potential was realized in 1990 aboard the Mir orbital station when the first successful production of fresh vegetables—specifically radishes and Chinese cabbage—in space was achieved. Subsequent experiments have successfully grown protein-rich wheat and beans using Balkanite (a specific medium based on Kardzhali clinoptilolite) and Balkanin technology [94].

Although there is currently no official European Union authorization for clinoptilolite-containing materials as novel food or food additives, there has been a global trend to use natural zeolites for home healthcare, either as a powdered nutritional supplement or as a water additive. However, the origin and processing of the zeolites used are not always entirely clear. The EU authorizes the use of sedimentary clinoptilolite as an animal feed additive if the raw material meets specific requirements, including a clinoptilolite content of at least 80% and a maximum content of 20% of other silicate minerals [95]. Initially implemented in Japan during the 1960s [96] and later in Bulgaria, the employment of natural zeolites, particularly clinoptilolite, has evolved into a core component of animal husbandry practices, attributable to its capacity to stimulate growth and augment overall productivity. Beyond enhancing performance, these minerals offer significant prophylactic benefits by reducing disease frequency and employing high sorption capacities to support the detoxification of living organisms.

The detoxification ability of Na-enriched clinoptilolite, applied as a food supplement in an ecotoxicological experiment involving conventional food and lead, was demonstrated in Ref. [96]. Modified NZ has been described as a protective barrier that prevents toxic contaminants from entering the human food chain in industrially polluted regions. This additive significantly inhibits lead absorption in the gastrointestinal tract because of its structural stability in acidic environments. It has also been demonstrated that it can reduce lead bioaccumulation in critical tissues (e.g., the liver and bones) by up to 90% through the process of effectively trapping heavy metals for subsequent excretion. Recent studies on clinoptilolite from the Beli Plast deposit further validate these benefits, showing that a 12.5% zeolite diet can boost mammalian body weight by 21% without inducing oxidative stress or gene toxicity. Ultimately, these findings confirm that modified zeolites from the North-Eastern Rhodopes may serve as a safe, high-capacity detoxification tool with significant potential for future pharmaceutical and nutritional applications [97].

Due to its high selectivity and capacity for Cs ions in removing contaminants from liquid waste, clinoptilolite has played a critical role in treating contaminated areas following the Chernobyl [98] and Fukushima [99] accidents. Its ability to selectively remove radioactive isotopes such as ^137^Cs and ^90^Sr makes clinoptilolite indispensable for ensuring the safety of food and water in nuclear-affected regions and for managing industrial nuclear effluents from water solutions [34]. In the early 90s, the first Bulgarian food supplement, sorbent CLS-5, was produced and approved for human use in the country. The distribution of the powder was intended to protect the population in the area of the Kozloduy Nuclear Power Station in the event of a radiation emergency [100]. A series of six modifications, incorporating food components, was subsequently developed based on CLS-5. In 2013, two types of food supplements were produced: Clinodetox and Clinodetox-Vita + 200. Additionally, KLS-10-MA has been developed for use in cases of chronic heavy metal poisoning in animals [96,101].

In 2013, after conducting extensive research in collaboration with experts, scientists and producers, a unique zeolite filter was developed. A zeolite water named “Sevtopolis” was produced using a complex system filtration process. Subsequently, a mixture of zeolite-filtered water and organic products was formulated, which is now available for commercial use. The effectiveness of zeolite water against Gram-positive and Gram-negative bacteria, as well as its potential use as a prophylactic and adjunctive therapy for bacterial infections, is reported in Ref. [102]. It is important to note that the use of natural zeolites may result in increased mineralization, despite their documented effectiveness in improving drinking water quality [103]. This is a crucial consideration that requires careful evaluation.

Metallic poisoning is an essential component of toxicology due to metals’ ubiquity and accessibility. The pathways of heavy metal ions in the human body have been well documented. They persist in the body, leading to chronic toxicity over time because of their slow excretion. Due to the risks of heavy metal bioaccumulation, energy-efficient zeolite ion-exchange methods—first used in the 19th century—remain the leading sustainable strategy for pollutant removal [12,104].

Despite extensive research on natural aluminosilicates for wastewater treatment and detoxification, recent studies increasingly focus on local minerals to explore their potential across a variety of application fields (Table 3).

While natural minerals are highly valued for their substantial ion-exchange properties, they do not exhibit the specific engineered characteristics of synthetic varieties. Synthetic production remains the only method for creating materials that are optimally suited to specialized industrial needs. A zeolite-based adsorbent capable of rapid, total dye absorption was produced from alkali-treated waste slag from the “Sviloza” Thermal Power Plant, located in Svishtov, Bulgaria. This transformed material leverages a superior active surface area to outperform raw slag in wastewater treatment tests. Moreover, specialized zeolites, such as Hydroxy Sodalite and Na-X, were produced from Bulgarian fly ashes from various thermal plants via hydrothermal conversion. These materials serve critical roles in nuclear safety by trapping radioactive isotopes, capturing CO_2_ for gas separation, and catalyzing the degradation of harmful volatile organic compounds. Another two synthetic types—NaA and Sodalite—were developed from rice husks and recycled aluminum cans through new alkali activation techniques [92]. In contrast, it has been demonstrated that natural clinoptilolite exhibits superior performance compared to synthetic zeolite L produced at University “Prof. Dr. Assen Zlatarov” in Burgas in the removal of Fe^2+^ and Mn^2+^ ions from aqueous solutions. It is noteworthy that NZ have demonstrated their potential as more expeditious and economically viable adsorbents for implementation in practical water treatment [115]. Eventually, the low cost and vast natural abundance of NZ make them viable materials for widespread industrial and environmental use [12].

Ultimately, these diverse applications demonstrate how Bulgaria has successfully advanced from basic resource extraction to experimental research activities, different modifications, and even the manufacturing of specialized products.

## 4. Conclusions

This review surveys selected literature on zeolites, emphasizing major advances in both fundamental and applied research on this important class of minerals. It also traces the evolution of natural zeolites into widely utilized industrial and experimental materials.

Mapping of the regional zeolite research continuum reveals a highly dynamic and rapidly evolving scientific landscape. The substantial number of publications produced over the past decade demonstrates the strength and sustained momentum of regional research activities, with original scientific articles accounting for more than 97% of the total academic output. On the global stage, the findings reaffirm Turkey’s position as a leading contributor to zeolite research and highlight Bulgaria’s steadily growing presence within the international scientific community in this field. Furthermore, international collaboration among researchers from diverse scientific disciplines is essential for overcoming current challenges in material processing and production, thereby accelerating the sustainable development and application of zeolites.

Nevertheless, several important challenges remain. These are primarily associated with (i) material purity; (ii) particle size, activation methods, and the nature of specific contaminants; and (iii) commercialization practices and misleading product labeling. Crucially, further clinical validation and more robust regulatory oversight remain essential for human biomedical applications.

## Figures and Tables

**Table 1:** Top 10 cited articles from Turkish and Bulgarian authors.

Author(s), Year	Country	Number of Citations	Study Objective/ NZ Application	Study Design	Sample Origin	Ref.
Shahmansouri, A. et al., 2021	Iran, Australia, China, Turkey	311/331 *	As a pozzolanic cement replacement in geopolymer concrete	Research article	Mined from quarries in Semnan, Iran	[36]
Eroglu, N. et al., 2017	Turkey, Greece	264/283 *	Summary of the main uses of NZ in food and agriculture	Review	Not applicable	[15]
Nikolov, A. et al., 2017	Bulgaria, Netherlands	143/147 *	Preparation of geopolymers based on NZ	Research article	From Beli Plast, Bulgaria	[37]
Yakar, A. et al., 2018.	Turkey, Czech Republic	128/131 *	As filtration media in wastewater treatment and bioelectric production	Research article	Not specified	[38]
Ates, A. and Akgül, G., 2016	Turkey	131/127 *	Determination of the adsorption capacity of NZ and modified NZ in manganese removal	Research article	From the Manisa-Demirci region, Turkey	[39]
Safarpour, M. et al., 2017	Iran, Turkey	103/104 *	As a hydrophilic zeolite material embedded in reverse osmosis membrane fabrication	Research article	From Mianeh region in the northwest of Iran	[40]
Sözen, A. et al., 2019	Turkey	89/91 *	Heat transfer enhancement of a heat pipe using aqueous clinoptilolite nanofluid	Research article	Not specified	[41]
Nikolov, A. et al., 2020	Bulgaria, Netherlands	85/88 *	As geopolymer precursor	Research article	From Beli Plast, Bulgaria	[42]
Kaplan, G. et al., 2021	Turkey	58/75 *	As partial cement substitute in durable cementitious composites	Research article	Not specified	[43]
Ayanoğlu, A. and Yumrutaş, R., 2016	Turkey	68/73 *	As an additive in pyrolysis process to upgrade waste tire oils into fuel-like products	Research article	Not specified	[44]

* Total citation counts as of May 2026.

**Table 2:** Experimental studies involving Turkish natural zeolite samples.

Deposit	Zeolitic Tuff Composition	Modification	Application	Ref.
Manisa-Gördes	up to 70% clinoptilolite	not reported	U(VI) removal	[58]
Manisa-Gördes	up to 70% clinoptilolite	not reported	Po removal	[59]
Manisa-Gördes	not specified	not reported	^137^Cs removal	[60]
Kutahya, Canakkale-Biga, Cankiri-Corum, and Manisa-Demirci regions	clinoptilolite, clinoptilolite and analcime, clinoptilolite and heulandit	not reported	Cs^+^ and Sr^2+^ removal	[61]
Bigadic	clinoptilolite-rich tuffs	chemical treatment	U(VI) removal	[62]
Manisa-Gordes	Up to 70% clinoptilolite	polyacrylamidoxime-modified	^210^Po	[63]
Polatlı (Ankara), Bigadic (Canakkale), Saphane (Balikesir), Gediz (Kutahya), and Gordes (Manisa)	75–98% clinoptilolite, 5–10% smectite, and 5–10% feldspar	not reported	^137^Cs, ^60^Co, ^90^Sr and ^110m^Ag	[64]
Dogantepe (Amasya)	45% clinoptilolite, 35% mordenite, 15% feldspar, and 5% quartz	not reported	NH_4_^+^ removal	[65]
Balıkesir	clinoptilolite	chemical treatment	NH_4_^+^ removal	[66]
Yıldızeli town of Sivas	95% clinoptilolite/heulandite and mordenite	not reported	NH_4_^+^ removal	[57]
Gördes and Bigadiç	clinoptilolite, k-feldspar, and quartz	not reported	Zn^2+^ removal	[67]
Gördes and Bigadiç	clinoptilolite	chemical activation	Pb, Zn, and Cd	[68]
Gördes and Bigadiç	clinoptilolite, k-feldspar, and quartz	chemical activation	ethylene removal	[69]
received from the Incal Company, Gördes	clinoptilolite	modification with quaternary amines	anionic azo dyes	[70]
received from the Incal Company, Gördes	clinoptilolite	modification with hexadecyl trimethyl ammonium bromide	Remazol Brilliant Blue R and Remazol Yellow reactive dyes	[71]
supplied by “Tusorb”	not specified	chitosan-based modification	Reactive Orange 122	[72]

**Table 3:** Experimental studies involving Bulgarian natural zeolite samples.

Deposit	Zeolitic Tuff Composition	Modification	Application	Ref.
Beli Plast and Beliya Bair	over 75% clinoptilolite	chemical activation	Pb^2+^, Cd^2+^, Fe^2+^ and Mn^2+^ adsorbents	[49]
Beli Plast and Golobradovo	clinoptilolitized pyroclastics	not reported	neutralizing water pH; metal and ion adsorption	[105]
Beli Plast	clinoptilolite	not reported	Fe^2+^, Pb^2+^, and Cu^2+^ adsorbents	[106]
Beli Plast	clinoptilolite	chemical activation	adsorption of Pb^2+^	[107]
Beli Plast	clinoptilolite	Co- and Mn-impregnation	catalyst carrier	[108]
Not specified	not specified	La and Fe incorporation	PO_4_^3−^	[2]
East Rhodopes	not specified	low-temperature plasma treatment	cationic dye removal	[109]
Beli Plast and Most Golobradovo	clinoptilolite	Ag-impregnation	as catalysts for decomposition of ozone	[110]
East Rhodopes	75% clinoptilolite	Ag-impregnation	antibacterial activity	[111]
East Rhodopes	76% clinoptilolite	Ag-impregnation	antibacterial activity	[48,112]
East Rhodopes	clinoptilolite	Ag-impregnation	biocompatibility and toxicity	[112]
East Rhodopes	clinoptilolite	Lavender essential oil impregnation	innovative bioactive products	[113]
Not specified	not specified	Lavender oil essence encapsulation	preserving the aroma of the oils	[114]

## Data Availability

No new data were created or analyzed in this study. Data sharing is not applicable.
